# Progress in Surgery

**Published:** 1901-11-09

**Authors:** 


### Progress in Surgery.

### RENAL SURGERY.

**Movable Kidney.**—G. M. Edebohls (N. Y.)[1] has written on the use of bandages for nephroptosis. Since operative fixation was proposed and carried out by Hahn in 1881, the advocates of the bandage treatment have become relatively few. Edebohls considers the use of belts, etc., to envelop and sustain the whole or a part of the abdomen to be rational, and that they are most satisfactory when the movable kidney is associated with general enteroptosis. They are not satisfactory when movable kidney produces symptoms in the absence of general enteroptosis. This applies also to the low-reaching corset. The writer, however, regards all appliances devised to exert direct pressure on the displaced organ by means of a special kidney pad as not only illusory but really harmful. It is impossible to maintain a movable kidney in place for any length of time by the hand with the patient upright, however intelligently applied. It must, therefore, be impossible for a mechanical contrivance to do it. It requires two hands, one placed behind and the other in front, to sustain the kidney. The pressure required could not be endured by any patient, and even if it could it would injure the kidney. Menge is quoted, who found that after diagnostic palpation in 15 out of 20 cases the urine contained albumin and a little blood, though before the palpation the secretion had been normal. The albumin was always found in greater quantity than could be accounted for by the blood present. Menge warns against massage of the kidney and against pressure being applied with pads. Edebohls next shows that his cases of nephropexy indicate that chronic interstitial nephritis is more common proportionately in patients with movable kidneys than in others. In 241 nephropexies performed upon 176 patients he met with 19 kidneys affected with chronic nephritis and two with acute nephritis. In the chronic cases the disease was unilateral in nine. In a large proportion of these cases the fixation of the kidney cured the nephritis, and the writer now suggests surgical intervention for the purpose of attempting to cure chronic nephritis whether the kidney is movable or not. In the latter case he would simply denude the kidney of its capsule. This will tend to establish a free collateral circulation. A further reason for not employing a kidney pad is based upon the nearly constant association of congestion of the vermiform appendix with movable right kidney. The writer, in 54 of his last 74 patients operated upon for movable kidney, also removed diseased appendices. In all cases in which relief cannot be obtained from a proper simple bandage or from a long, and low-reaching corset, nephropexy is indicated. Arbuthnot Lane[2] draws attention to the effect that thoracic incapacity, so frequently found in girls and women, has in causing the kidneys to occupy a lower position in the body than normal. The individuals are usually thin, flat-chested, round-shouldered, and stooping, and have a prominent abdomen. A good many of them suffer from pain in the loin, and the kidney is often tender on pressure. The writer has known the kidney, during an expulsive effort, to fall down into the true pelvis and cause the most violent and painful straining during defœcation. Lane believes the chief cause of pain in movable kidney to be the engorgement of the organ from interference with the return of blood into the vena cava. This makes the organ swollen, tense, and painful. Even walking may be painful, and the hip-joint kept stiff to diminish the pain. A moderate mobility in one patient may cause very great pain, while in another free mobility may be present without pain. Partial or complete occlusion of the ureter he considers to be rare. An obstruction of the upper part of the ascending colon by adhesions is said to be common and to be frequently mistaken for movable kidney or for stone in the kidney. The condition produces distension and hypertrophy of the cæcum. The intimate fixation of the colon to the kidney in these cases leads to pain closely simulating that of renal origin. Separation of the kidney from its surrounding structures may cure these cases. The author has never known a kidney become loose again after fixation by his method. A. H. Goelet[3] writes that palliative measures are of no avail if the degree of prolapse of the kidney is sufficient to produce symptoms. Mobility is found in one in every four or five of the women who come for gynæcological treatment, and half of these cases require operation. The emaciation often associated with the condition is probably often entirely secondary and due to the digestive and nervous disturbances which are present. Often, however, the patients have not lost flesh and are not thin. Movable kidney is as frequent in women who have never borne children as in those who have, and is often found in young girls. Excessive vomiting may be considered a cause, and whooping cough. Chronic digestive disturbance with intestinal distension are the most frequent symptoms. Pain is often referred to the ovarian region. Pain in the kidney region itself is infrequent. The author has found that the best method of examination is to have the patient standing and leaning slightly forwards and then to grasp the loin with the fingers and thumb of one hand, the thumb being in front and sunk well into the abdominal wall just below the border of the ribs at the moment when expiration is complete. If out of position the kidney must be below the thumb and can be pressed up under the thumb by the other hand laid flat on the abdominal wall. In operating Goelet passes the sutures in a special way. The needle is first inserted rather superficially on the lateral surface of the lower half of the exposed kidney from above downward; then it is inserted deeply and transversely, and again superficially in the opposite lateral surface from below upward. The object is to distribute the strain on the central or transverse insertion and lessen the chance of it tearing out. Two silkworm gut sutures are passed in the manner described, one above the other, and the ends are carried through the muscles and skin near the upper angle of the wound and tied over a strip of gauze. The sutures are removed in a fortnight. A. Sturmdorf (N.Y.)[4] criticises adversely the technique of the operation for movable kidney recently described by R. T. Morris. The latter advocated stripping a large quadrilateral flap of the capsule from the posterior surface of the kidney, leaving it attached at the convex border, and drawing this flap through a slit in the psoas or quadratus muscle and suturing it there. Sturmdorf considers the flap too large, and hence likely to disintegrate; also that the base of the flap forms a hinge joint at the convex border of the kidney which will favour a swinging movement of the other border of the organ forward and outward. To avoid this the writer makes two flaps of the same part of the capsule that Morris utilised, turning up one towards the convex border and the other towards the concave border. The two flaps are drawn through slits in the adjacent muscular tissue. In addition, two trans-paranchymatous fixation sutures may be passed and fixed externally, and three other buried ones used. Tubby[5] remarks that if there is any disturbance of the kidney at all, either stone, movable kidney, or hydronephrosis, almost always there are associated certain disturbances of the colon with flatulence and pain. He considers that no patients should be operated upon until they have tried a properly fitting abdominal belt for three months. In operating the usual procedure is said to be that of passing seven or eight sutures through the renal substance. It is interesting to find that I. Israel, as shown in his recent book on kidney diseases,[6] considers that very few cases of movable kidney require operative treatment. He believes that in many, perhaps in most cases, symptoms are attributed to the movable kidney which are really due to other causes, namely, to neuropathic conditions and disorders of the generative organs. He reports only seven cases of simple nephropexy.

[1] Med. Rec., May 4, 1901, p. 690. [2] Clin. Journ., June 5, 1901, p. 97. [3] Med. Rec., June 1, 1901. p. 841. [4] Med. Rec., June 22, p. 998. [5] Clin. Journ., May 29, 1901, p. 87. [6] *Vide* Centralb. f. Chir., July 27, 1901.

